# Supplementary material for: Genetic variation across trophic levels: A test of the correlation between population size and genetic diversity in sympatric desert lizards
Source: PLoS One. 2019 Dec 5;14(12):e0224040. doi: 10.1371/journal.pone.0224040 (PMC6894812; doi:10.1371/journal.pone.0224040)
Supplement: S1 Table — Collection locality for each lizard sampled, by species. (DOCX) [file pone.0224040.s001.docx]

**S1 Table. Specimen collection localities.**

| ***Crotaphytus bicinctores* collection details** | | |  |
| --- | --- | --- | --- |
| **Individual ID** | **Site Name** | **Decimal Degrees North** | **Decimal Degrees West** |
| 047 | Lava | 35.20105556 | -115.86805556 |
| 049 | Lava | 35.20336111 | -115.86966667 |
| 149 | Lava | 35.19711111 | -115.86247222 |
| 459 | Zzyzx | 35.16902778 | -116.10797222 |
| 460 | Zzyzx | 35.16500000 | -116.10722222 |
| 461 | Zzyzx | 35.16458333 | -116.10716667 |
| 465 | Lava | 35.20069444 | -115.86758333 |
| 471 | Lava | 35.20011111 | -115.86811111 |
| 472 | Lava | 35.19850000 | -115.87075000 |
| 474 | Lava | 35.20069444 | -115.86780556 |
| 476 | Zzyzx | 35.16430556 | -116.10825000 |
| 482 | Zzyzx | 35.16438889 | -116.10722222 |
| 484 | Lava | 35.19963889 | -115.86619444 |
| 489 | Zzyzx | 35.16560000 | -116.10730000 |
| 494 | Lava | 35.20191667 | -115.87028333 |
| 496 | Zzyzx | 35.16583333 | -116.10730000 |
| 508 | Zzyzx | 35.15400000 | -116.10608333 |
| 509 | Zzyzx | 35.14488889 | -116.11080556 |
| 510 | Zzyzx | 35.17241667 | -116.10968333 |
| 511 | Zzyzx | 35.16473333 | -116.10721667 |
| 517 | Lava | 35.20094444 | -115.86677778 |
| 518 | Zzyzx | 35.16475000 | -116.10719444 |
| 519 | Zzyzx | 35.17305556 | -116.11013889 |
| 520 | Zzyzx | 35.17388889 | -116.11041667 |
| 521 | Zzyzx | 35.14269444 | -116.10844444 |
| 522 | Zzyzx | 35.17327778 | -116.11041667 |
| 526 | Zzyzx | 35.15334444 | -116.10665556 |
| 527 | Zzyzx | 35.15400278 | -116.10610000 |
| 528 | Zzyzx | 35.14272222 | -116.10413889 |
| 561 | Lava | 35.19966667 | -115.86550000 |
| 575 | Zzyzx | 35.16427778 | -116.10725000 |
| 576 | Zzyzx | 35.16572222 | -116.10722222 |

| ***Gambelia wislizenii* collection details** | | |  |
| --- | --- | --- | --- |
| **Individual ID** | **Site Name** | **Decimal Degrees North** | **Decimal Degrees West** |
| 051 | Lava | 35.19708333 | -115.87038889 |
| 056 | Lava | 35.18091667 | -115.84919444 |
| 070 | Lava | 35.19955556 | -115.87305556 |
| 087 | Lava | 35.20494444 | -115.87086111 |
| 104 | Lava | 35.19150000 | -115.86143333 |
| 155 | Lava | 35.20861111 | -115.87500000 |
| 161 | Lava | 35.18425000 | -115.76747222 |
| 214 | Lava | 35.21800000 | -115.73672222 |
| 261 | Lava | 35.21455556 | -115.87458333 |
| 283 | Lava | 35.15641667 | -115.79000000 |
| 286 | Lava | 35.17113889 | -115.77911111 |
| 313 | Lava | 35.25655556 | -116.03886111 |
| 316 | Lava | 35.21736111 | -115.86236111 |
| 362 | Lava | 35.20600000 | -115.75925000 |
| 378 | Lava | 35.23783333 | -115.72352778 |
| 424 | Kelso | 34.89502778 | -115.70283333 |
| 432 | Kelso | 34.88963889 | -115.71625000 |
| 490 | Lava | 35.20066667 | -115.86818333 |
| 495 | Lava | 35.19933333 | -115.85966667 |
| 506 | Lava | 35.17997222 | -115.77197222 |
| 507 | Kelso | 34.89502778 | -115.68752778 |
| 516 | Lava | 35.20230556 | -115.76277778 |
| 524 | Kelso | 34.89822222 | -115.66763889 |
| 530 | Lava | 35.18311111 | -115.76936111 |
| 531 | Lava | 35.18169444 | -115.85519444 |
| 532 | Lava | 35.18527778 | -115.86355556 |
| 539 | Lava | 35.22619167 | -115.88013056 |
| 555 | Kelso | 34.88631944 | -115.72195556 |
| 569 | Lava | 35.18355556 | -115.76844444 |
| 570 | Lava | 35.18291667 | -115.76858333 |
| 572 | Lava | 35.19519444 | -115.86825000 |
| 573 | Lava | 35.19430556 | -115.86136111 |
| 582 | Lava | 35.19816667 | -115.87052778 |
