# Supplementary material for: Genetic variation across trophic levels: A test of the correlation between population size and genetic diversity in sympatric desert lizards
Source: PLoS One. 2019 Dec 5;14(12):e0224040. doi: 10.1371/journal.pone.0224040 (PMC6894812; doi:10.1371/journal.pone.0224040)
Supplement: S1 Fig — Haplotype networks for all species, showing the haplotypes by collection locality. Within each network, circles are proportionate to number of copies of a haplotype (networks not on same scale). Hash marks between haplotypes represent the number of mutational steps. (PDF) [file pone.0224040.s003.pdf]

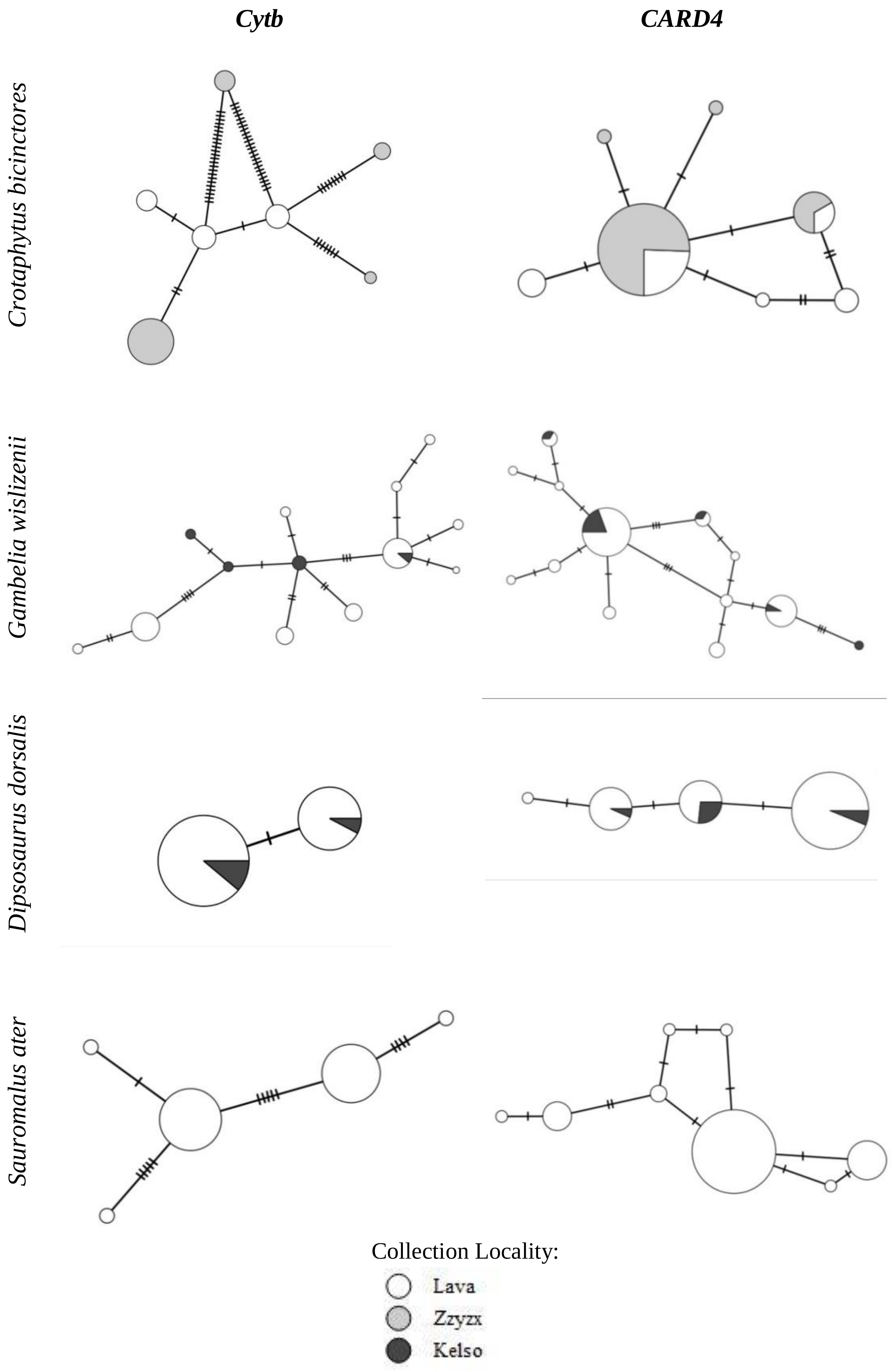

Cytb
CARD4
Crotaphytus bicinctores
Gambelia wislizenii
Dipsosaurus dorsalis
Sauromalus ater
Collection Locality:
Lava
Zzyzx
Kelso

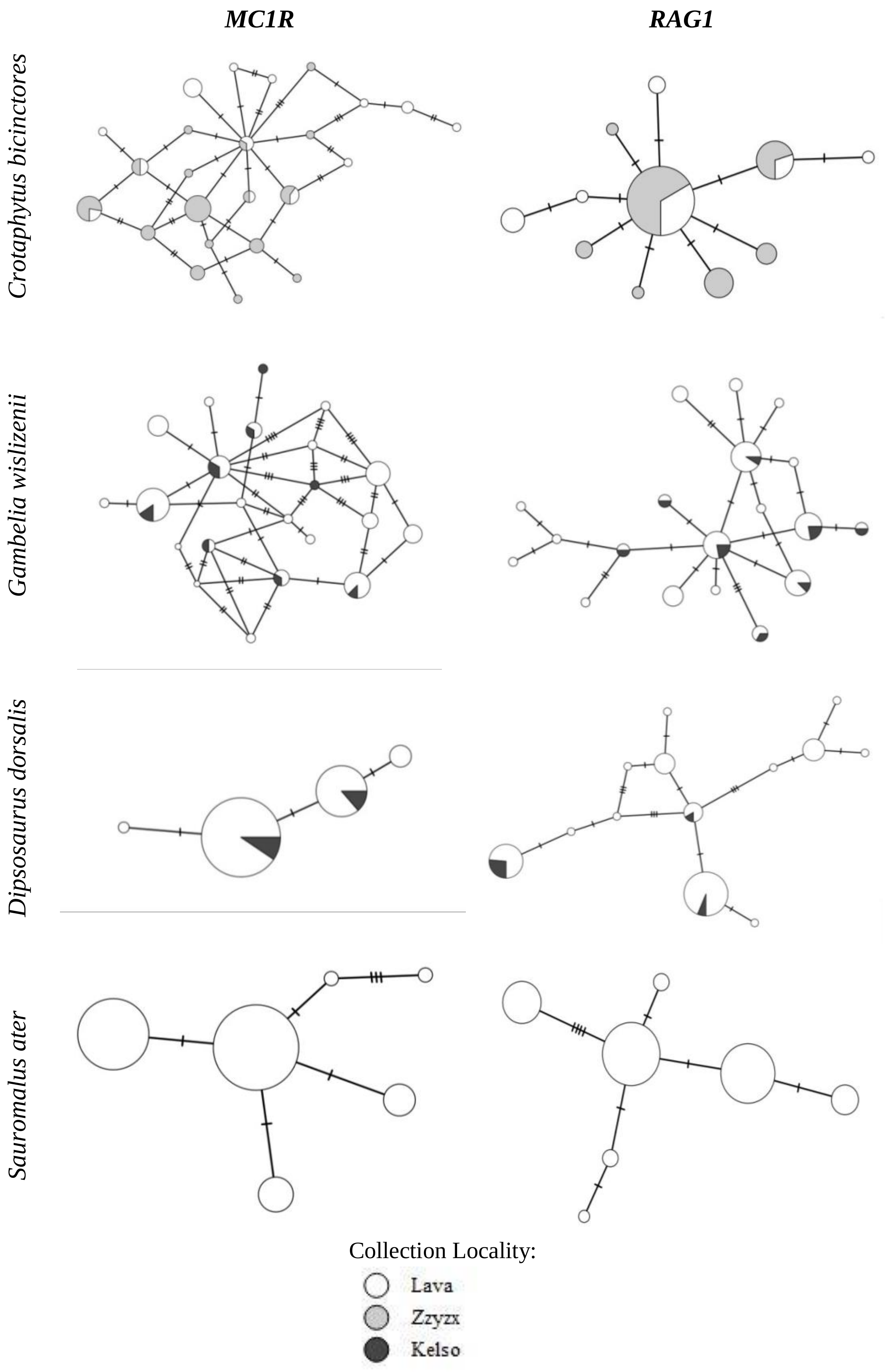
MC1R
RAG1
Crotaphytus bicinctores
Gambelia wislizenii
Dipsosaurus dorsalis
Sauromalus ater
Collection Locality:
Lava
Zzyzx
Kelso
