# Supplementary material for: Genetic variation across trophic levels: A test of the correlation between population size and genetic diversity in sympatric desert lizards
Source: PLoS One. 2019 Dec 5;14(12):e0224040. doi: 10.1371/journal.pone.0224040 (PMC6894812; doi:10.1371/journal.pone.0224040)
Supplement: S1 File — (DOCX) [file pone.0224040.s004.docx]

**S1 File. Supplementary methods – Primers and PCR protocols.**

Sequencing primers for all other locus-species combinations were the same as PCR primers.

| **PCR Protocols (continued)** | |  |  |  |
| --- | --- | --- | --- | --- |
| **Program Name** | **Step** | **Temperature (°C)** | **Time** | **Number of Cycles** |
| RAG1 | Initial Denaturation | 94° | 5:00 | 1 |
|  | Denaturation | 94° | 0:30 | 5 |
|  | Annealing | 52° | 1:30 |  |
|  | Extension | 72° | 1:00 |  |
|  | Denaturation | 94° | 0:30 | 41 |
|  | Annealing | 50° | 1:30 |  |
|  | Extension | 72° | 1:00 |  |
|  | Final Extension | 72° | 5:00 | 1 |
|  | Refrigeration | 5° | Inf. |  |
| MC1R_2 | Initial Denaturation | 94° | 5:00 | 1 |
|  | Denaturation | 94° | 0:30 | 5 |
|  | Annealing | 52° | 1:30 |  |
|  | Extension | 72° | 1:00 |  |
|  | Denaturation | 94° | 0:30 | 35 |
|  | Annealing | 50° | 1:30 |  |
|  | Extension | 72° | 1:00 |  |
|  | Final Extension | 72° | 5:00 | 1 |
|  | Refrigeration | 5° | Inf. |  |
