# Supplementary material for: Genetic variation across trophic levels: A test of the correlation between population size and genetic diversity in sympatric desert lizards
Source: PLoS One. 2019 Dec 5;14(12):e0224040. doi: 10.1371/journal.pone.0224040 (PMC6894812; doi:10.1371/journal.pone.0224040)
Supplement: S2 File — (DOCX) [file pone.0224040.s005.docx]

**S2 File. Randomization R Script**

This script requires a .fasta input file containing the haplotype pairs to be randomized. This should be created from a PHASE .out_pairs file. The heading for each haplotype needs to be in the form >Individual number;a or b; pair number; probability determined by PHASE (for example, “>047;a;1;0.015”). The script will produce a specified number of .arp files along with a .arb batch file for Arlequin, so that all can be run together as a batch and summarized.

#this code will be the number of genes in the Arlequin file header

genenum<-readline("How many genes (chunks of DNA) were sequenced? ")

#this code creates the Arlequin header

arlheader<-'[Profile]

Title =""

NbSamples = 1

DataType = DNA

GenotypicData = 0

LocusSeparator = WHITESPACE

MissingData = "-"

GameticPhase = 0

RecessiveData = 0

[Data]

[[Samples]]

SampleName = "pop_1"'

endofheader1<-"SampleSize = "

endofheader2<-"SampleData = {"

footer1<-paste("SampleSize = ",genenum)

footer2<-"SampleData = {"

#arlequin.header<-c(arlheader,"\n",footer1,"\n",footer2,"\n")

arlequin.header<-c(arlheader,footer1,footer2)

#read in fasta file with seqinR package

outfile.name<-readline("What do you want to call the output files (no numbers, just the base name)? ")

random.total<-readline("How many randomizations do you want? ")

random.vector<-c(1:random.total)

for (z in random.vector){

workingfile<-"WORKING DIRECTORY/FILE NAME.fasta"

CMpractice <- system.file (workingfile ,package = "seqinr")

a<-read.fasta (file = workingfile , as.string=TRUE, set.attributes = TRUE)

B<-read.fasta (file = workingfile , as.string=TRUE, set.attributes = FALSE)

#to split header name, each part wanted needs to be delimited by a ";" for this syntax, can also do _ or () or whatever

Header<-strsplit(attr(B,"name"), ";")

#identify each part of the name

HedMat<-matrix(unlist(Header), ncol=4, byrow=TRUE)

#df: as.data.frame and give columns names

HedDF<-as.data.frame(HedMat)

colnames(HedDF)<- c("IND","A/B","Pair","Freq")

#create "vector" lists with values from the column from df matrix

indiv<-HedDF[,1]

AB<-HedDF[,2]

Pair<-HedDF[,3]

Freq<-HedDF[,4]

#data.frame to add column a

#G<- data.frame(indiv, AB, Pair, Freq, a)

# isnt working yet

#Trying now with B

D<-unlist(B, use.name=F)

#making all values vectors and combining into ONE matrix/data frame

Full<- data.frame(as.vector(indiv), as.vector(AB), as.vector(Pair), as.vector(Freq), D)

Full[,5]=as.character(Full[,5])

#rename columns

colnames(Full)<-c("IND","A.B","Pair","Freq", "Sequence")

#finding if sequence has an N or not

grepl("n", Full[29,5], ignore.case=F)

#generate list of all pairs to compare

pairs<- combn(as.numeric(rownames(Full)),2)

n.pairs<-dim(pairs)[2]

#create character array from sequences

Seq<-strsplit(Full[,5],"")

#create list of haplotypes and add sequence 1 to haplo list

haplo.list<-rep(0,dim(Full)[1])

haplo.list[1]<-1

next.haplo<-2

#haplo.list<-c(haplo.list, Full[1,5])

#loop for pair comparisons; loop through each comparision pair

for(i in 2:dim(Full)[1]){

#iterate through each sequence between the first and current (i)

for(j in 1:(i-1)){

#n-check

if(sum(grepl("n",c(Full[i,5],Full[j,5])))>0){

#grab array at index i

i.array<-Seq[[i]]

i.ns<-which(i.array=="n")

#grab array at index j

j.array<-Seq[[j]]

j.ns<-which(j.array=="n")

temp.i<-i.array

temp.j<-j.array

temp.i[j.ns]<-"n"

temp.j[i.ns]<-"n"

if(all(temp.i==temp.j)){

haplo.list[i]<-haplo.list[j]

break

}

}

else{

if(Full[i,5]==Full[j,5]){

haplo.list[i]<-haplo.list[j]

break

}

}

}

if(haplo.list[i]==0){

haplo.list[i]<-next.haplo

next.haplo<-next.haplo+1

}

}

updated.matrix<-cbind(Full, haplo.list)

# '$' references a name: so I can recall a particular variable. for example: updated.matrix$haplo.list

#Now for the real stuff

#randomization of selection of an individuals haplotype and output to a fasta file

#getting Freq's and IND's to not be factors and grabbing ones that are larger than .7

updated.matrix$Freq<-as.numeric(as.character(updated.matrix$Freq))

# updated.matrix$IND<-as.numeric(as.character(updated.matrix$IND))

updated.matrix$Pair<-as.character(updated.matrix$Pair)

#this tells me all individuals in my file

indlist<-levels(as.factor(updated.matrix$IND))

#"length" makes sure you take into account each value, finds the amount of values in a list

#anything with parentheses indicates parameters..."else" doesnt allow parameters

Final<-data.frame()

for(x in 1:length(indlist)){

temp.ind<-updated.matrix[updated.matrix$IND==indlist[x],]

if(any(temp.ind$Freq>=.7)){

Final<-rbind(Final,temp.ind[temp.ind$Pair==(temp.ind$Pair[temp.ind$Freq>=.7][1]),])

} else{

pairlist<-levels(as.factor(temp.ind$Pair))

random<-sample(pairlist,1)

Final<-rbind(Final, temp.ind[temp.ind$Pair==random,])

}

}

Final

filename<-paste(outfile.name,sep="",z,".arp")

write(arlequin.header,filename,append=T)

for(y in 1:length(Final$IND)){

fasta.name<-paste(Final$IND[y],Final$A.B[y],"_",Final$Pair[y],"(",Final$Freq[y],")_",Final$haplo.list[y],sep="","\t"," 1","\t")

final.entry<-paste(fasta.name,Final$Sequence[y])

write(final.entry,filename,append=T)

}

write("}",filename,append=T)

batch.file.name<-paste(outfile.name,sep="",".arb")

write(filename,batch.file.name,append=T)

}
