# Supplementary material for: Genetic variation across trophic levels: A test of the correlation between population size and genetic diversity in sympatric desert lizards
Source: PLoS One. 2019 Dec 5;14(12):e0224040. doi: 10.1371/journal.pone.0224040 (PMC6894812; doi:10.1371/journal.pone.0224040)
Supplement: S3 File — (DOCX) [file pone.0224040.s006.docx]

**S3 File. R script for calculation of F_ST_ and Jost’s D.**

This script requires a .csv input file with three columns: the first labelled “Pop”, which identifies the subpopulation each individuals is from (A or B), and the second and third labelled “H1” and “H2”, containing the two haplotype numbers for each individual (for autosomal loci). There should be a row for each individual, and they must be sorted by subpopulation. Note that for simplicity, this script assumes there are exactly two subpopulations.

library("plyr")

data <- read.csv("FILE NAME.csv")

Find total number of individuals sampled from each subpopulation, then create a vector for each subpopulation with the counts of each haplotype. Then convert the counts to frequencies.

hap_numbers <- c(1:max(data$H1, data$H2))

hap_listA <- c(data$H1[1:nA], data$H2[1:nA])

df_hapA <- data.frame(haplotype = factor(hap_listA, hap_numbers))

hap_count_A <- as.vector(table(df_hapA$haplotype))

hap_listB <- c(data$H1[(nA+1):(nA+nB)], data$H2[(nA+1):(nA+nB)])

df_hapB <- data.frame(haplotype = factor(hap_listB, hap_numbers))

hap_count_B <- as.vector(table(df_hapB$haplotype))

hap_freq_A <- hap_count_A/(nA*2)

hap_freq_B <- hap_count_B/(nB*2)

Use vectors of haplotype frequencies in the equations from Jost (2008).

Ntilda <- 1/(mean(c((1/(nA*2)), (1/(nB*2)))))

H.A <- 1 - sum(hap_freq_A^2)

H.B <- 1 - sum(hap_freq_B^2)

H.S <- (1/2)*(H.A+H.B)

H.T <- 1 - (sum(((1/2)*(hap_freq_A + hap_freq_B))^2))

HS_est <- (2*Ntilda/(2*Ntilda -1))*H.S

HT_est <- H.T + (HS_est)/(2*Ntilda*2)

Dest <- ((HT_est-HS_est)/(1-HS_est))*(2/(2-1))

Calculate G_ST_ (adjusted for sample frequencies, rather than population frequencies) and Δ_ST_ (Jost 2008)_._

J_T <- sum((hap_freq_A/2 + hap_freq_B/2)^2)

J_S <- ((1/2)*(sum(hap_freq_A^2) + sum(hap_freq_B^2)))

Delta_ST <- J_S / J_T

GST_est <- (HT_est - HS_est)/HT_est

Create a table which displays the results. The value here called “GST_est” is reported elsewhere as F_ST_ (Nei 1973).

results <- c(HS_est, HT_est, GST_est, Dest, Delta_ST)

names(results) <- c("HS_est", "HT_est", "GST_est", "Dest", "Delta_ST")

results

The script for haploid datasets is very similar. However, it requires an input file with only two columns, one to identify the subpopulation (“Pop”) and one with the haplotype number for the individual (“H1”).

data <- read.csv("FILE NAME.csv")

nA <- (count(data, "Pop")[1,2])

nB <- (count(data, "Pop")[2,2])

hap_numbers <- c(1:max(data$H1))

hap_listA <- c(data$H1[1:nA])

df_hapA <- data.frame(haplotype = factor(hap_listA, hap_numbers))

hap_count_A <- as.vector(table(df_hapA$haplotype))

hap_listB <- c(data$H1[(nA+1):(nA+nB)])

df_hapB <- data.frame(haplotype = factor(hap_listB, hap_numbers))

hap_count_B <- as.vector(table(df_hapB$haplotype))

hap_freq_A <- hap_count_A/(nA)

hap_freq_B <- hap_count_B/(nB)

Ntilda <- 1/(mean(c((1/(nA)), (1/(nB)))))

H.A <- 1 - sum(hap_freq_A^2)

H.B <- 1 - sum(hap_freq_B^2)

H.S <- (1/2)*(H.A+H.B)

H.T <- 1 - (sum(((1/2)*(hap_freq_A + hap_freq_B))^2))

HS_est <- (Ntilda/(Ntilda -1))*H.S

HT_est <- H.T + (HS_est)/(Ntilda*2)

Dest <- ((HT_est-HS_est)/(1-HS_est))*(2/(2-1))

The script for G_ST_, Δ_ST_, and the output table are the same for haploid datasets as for diploid.
