# Supplementary material for: Genetic variation across trophic levels: A test of the correlation between population size and genetic diversity in sympatric desert lizards
Source: PLoS One. 2019 Dec 5;14(12):e0224040. doi: 10.1371/journal.pone.0224040 (PMC6894812; doi:10.1371/journal.pone.0224040)
Supplement: Attachment — Submitted filename: PONE-D-19-14658_reviewer.docx [file pone.0224040.s007.docx]

This is an interesting study that explores the relationship between population size and genetic diversity with four sympatric species- two herbivorous and two carnivorous lizards. I have a few broad and some specific comments.

Introduction

Generally, the introduction seems to be guided by the results that the authors got. If the motivation behind the study was to test whether neutral or selective forces shape genetic variation, this system and study design may not be most suited to address that.

In the introduction, the authors could focus more on the factors affecting genetic diversity in natural populations. It would be useful to have more information about both the intrinsic and extrinsic factors that affect genetic diversity. Authors mention the intrinsic factors of body size, mutation rates, generation times, etc. But extrinsic factors, such as extent and continuity of available habitat, do not find any mention.

66-67 Please elaborate on/ give examples of the confounding variables that you mention

Methods

91-92 It would be good to mention the body sizes here. People unfamiliar with the species and the region might find it useful.

Results

It would be nice to have a map showing study area with sample locations of the different species in different colours

It would be nice to compare generalist and specialist species within a trophic level instead of pooling them across the two levels as done in table 3.

A lot of information in the first paragraph under the ‘Mitochondrial DNA’ subheading can be moved to methods.

283-288 This information should go under the collection section in methods. Please also mention the distance between the different collection sites. Why was only one species sampled from Zzyzx? Other species do not occur in that area or were they not sampled for a reason?

Discussion

361-363 ‘The generalist species would be predicted to have a larger population size, since more usable habitat is available to them, and therefore they should have higher genetic diversity under the neutral theory. However, the data show no evidence for this pattern either.’ The authors cannot ignore the trophic level while making this statement. It is not okay to club the carnivore and herbivore generalists since population size is impacted by a combination of both- specialization and trophic level. Does the statement imply that the generalist herbivore in their study had a larger population size than the specialist carnivore? If yes, then its okay to club them in the same category and comment on expectations based on neutral theory. Whereas if they expect the population size in the following order: Carnivore specialist< carnivore generalist< herbivore specialist< herbivore generalist, the above statement cannot be made. The authors say nothing about the order in which they expect the population sizes to be for their study species. It should be explicitly mentioned in the introduction.

*G. wislizenii* has the lowest genetic differentiation and the highest genetic diversity based on multiple estimators. Does it also, among the study species, have a more continuous distribution and larger range?

Grundler et al (2019) does not find a reference in your paper. This recent study looks at the relationship between genomic diversity and abundance, occupancy and habitat specialization using genomic data from 30 species of lizards from arid Australia.
