# Supplementary material for: Genetic variation across trophic levels: A test of the correlation between population size and genetic diversity in sympatric desert lizards
Source: PLoS One. 2019 Dec 5;14(12):e0224040. doi: 10.1371/journal.pone.0224040 (PMC6894812; doi:10.1371/journal.pone.0224040)
Supplement: Attachment — Submitted filename: PLOS_review_trophic_gendiv.docx [file pone.0224040.s008.docx]

This study presents valuable data on genetic diversity, with an interesting ecological component that seeks to shed light on the still equivocal relationship between heterozygosity and population size. As such, it represents an important contribution to the field. However, there are several major points that I feel need reworking. My recommendation is publication after major revision, which should address the following issues:

1. The bulk of the data are presented in tables, which in my opinion lessens both the digestibility and impact of the results. The single figure that is presented could be improved with a key that indicates population size category of each of the species, such that readers can immediately pair heterozygosity with abundance/trophic category.
2. There are issues with the presentation of abundance data, which I feel impedes reproducibility of the study. The abundance data on which the authors base population size classification include a census study that I was unable to access; observational anecdotes (albeit over a long period of time); and coarse proxies. Proxies can be both necessary and valuable, but should be supported by more data or literature reference than found here. Similarly, observations of abundance should be supported with stronger justification as is done in Hague and Routman (2016) (frequently referenced in the current manuscript).
3. Because of the above two issues, I found it necessary to read Hague and Routman (2016) in conjunction with the manuscript in question. Much of the data and ecological context for the current study is presented in the 2016 manuscript. The two studies together present an interesting picture of the ambiguity of the relationship between genetic diversity and population size, and highlight the need for further study of ecological traits and genetic diversity. The current study seems to be a logical continuation of the 2016 project, and Figure 1 is a good example of the integration of data from each of these projects. However, the current manuscript could be improved by elaborating on the combined results in the discussion.

Specific comments:

L41-43 – what are the hypotheses for deviations from expectation in these studies? Good to have context here about why we might not expect things to correlate

L59 – not sure about the use of “haphazard” here; larger scale analyses are important for other reasons, especially considering impacts of population connectivity on genetic diversity

L117-118 – references for this?

L118-120 – References for census studies are inaccessible. I feel more detail is needed here to justify the validity of qualitative data – observations on abundance across seasons? Over periods of how long? Etc. Even a figure plotting the cited census data with genetic diversity would be valuable.

L124 – how large was the main collection area, and what are overall geographic ranges like for these species? More detail on connectivity of these species with surrounding populations would be useful when considering genetic diversity and population size (especially when there are deviations from expectation as we have here)

L147-174 – good use of alternate tests and simulations to validate analyses

L160-162 – there are standard statistical tests to quantify significant difference in pairwise measures that are sounder than a decision made by visual inspection. The citation for this method comes from a psychology article that I am unable to access, and seems, based on the abstract, more relevant for gaining inference from figures and improving research communication in a field that operates under different methods standards.

L187:

• Table 1 - Dn/ds statistic could be presented as a single column rather than or in addition to #synonymous and #nonsynonymous mutations in separate columns (which makes interpretation of this metric a two-step rather than one-step process)

• Tables summarized into figures would be helpful, especially Table 3. Much of this appears in fig 1, but the summary of information could be improved; cannot immediately tell which are small pops, which are large pops, which trophic level the species represent, etc. It’s not a lot to keep track of or to find in the text, but figures should be interpretable by themselves.

L236 - Tables 4 and 5 are identical

L334 – references for eco data? Found in open habitat doesn't necessarily = generalist…if rocky habitat is common then rock specialists could also be common…”small fraction of total habitat” – any kind of quantification available? What is the rest of the habitat characterized by? Ecological specialization is a very interesting and important consideration, and a valuable addition to the study, but it does feel a little too coarse as it stands. It would be helpful to at least include some references to strengthen inferences made here.

L386-387 - Reasoning behind “likely lack of statistical power”? Authors have a good number of individuals - do they mean due to number of species? Could use a little clarification

L395 – need some background here to contextualize coalescent variance

L407 – It would be valuable to situate the results of this study better in the context of previous results – Hague and Routman (2016) showed clear relationship between diversity and pop size but now we have conflicting relationship when we throw smaller pops into the mix – what could be going on here? This is especially interesting and merits additional study with all species to look for ecological correlations – and so should be mentioned with more detail in the discussion
